# Predictors of mortality among inpatients with COVID-19 infection in a tertiary referral center in the Philippines

**DOI:** 10.1016/j.ijregi.2022.07.009

**Published:** 2022-07-14

**Authors:** Anna Flor G. Malundo, Cybele Lara R. Abad, Maria Sonia S. Salamat, Joanne Carmela M. Sandejas, Jonnel B. Poblete, Jose Eladio G. Planta, Shayne Julieane L. Morales, Ron Rafael W. Gabunada, Agnes Lorrainne M. Evasan, Johanna Patricia A. Cañal, Julian A. Santos, Jeffrey T. Manto, Maria Elizabeth P. Mercado, Raniv D. Rojo, Eric David B. Ornos, Marissa M. Alejandria

**Affiliations:** a Division of Infectious Diseases, University of the Philippines – Philippine General Hospital, Taft Avenue, Ermita, Manila, National Capital Region, Philippines; b Division of Infectious Diseases, University of the Philippines – Philippine General Hospital, Manila, Philippines; c Department of Medicine, University of the Philippines – Philippine General Hospital, Manila, Philippines; d Department of Radiology, University of the Philippines – Philippine General Hospital, Manila, Philippines; e Department of Clinical Epidemiology, Faculty of Medicine and Surgery, University of Santo Tomas, Manila, Philippines; f College of Medicine, University of the Philippines, Manila, Philippines

**Keywords:** COVID-19, Philippines, epidemiology, mortality

## Abstract

**Objectives:**

The aim of this study was to determine the predictors of mortality and describe laboratory trends among adults with confirmed COVID-19.

**Methods:**

The medical records of adult patients admitted to a referral hospital with COVID-19 were retrospectively reviewed. Demographic and clinical characteristics, and laboratory parameters, were compared between survivors and non-survivors. Predictors of mortality were determined by multivariate analysis. Mean laboratory values were plotted across illness duration.

**Results:**

Of 1215 patients, 203 (16.7%) had mild, 488 (40.2%) moderate, 183 (15.1%) severe, and 341 (28.1%) critical COVID-19 on admission. In-hospital mortality was 18.2% (0% mild, 6.1% moderate, 15.8% severe, 47.5% critical). Predictors of mortality were age ≥ 60 years, COPD, qSOFA score ≥ 2, WBC > 10 × 10^9^/L, absolute lymphocyte count < 1000, neutrophil ≥ 70%, PaO_2_/FiO_2_ ratio ≤ 200, eGFR < 90 mL/min/1.73 m^2^, LDH > 600 U/L, and CRP > 12 mg/L. Non-survivors exhibited an increase in LDH and decreases in PaO_2_/FiO_2_ ratio and eGFR during the 2nd–3rd week of illness.

**Conclusion:**

The overall mortality rate was high. Predictors of mortality were similar to those of other reports globally. Marked inflammation and worsening pulmonary and renal function were evident among non-survivors by the 2nd–3rd week of illness.

## Highlights

• Mortality data were comparable to those of early reports relating to the wild-type SARS-CoV-2.
• Clinical and laboratory monitoring is critical during the 2nd to 3rd week of illness.
• Common and inexpensive laboratory tests may aid in the monitoring of patients.
• Clinical pathways can be adapted to local data, especially in resource-poor settings.

## INTRODUCTION

Coronavirus disease 2019 (COVID-19) has led to more than 547 million confirmed cases and 6.3 million deaths worldwide (World Health Organization, 2022). The Philippines is one of the COVID-19 hotspots in the Western Pacific Region, having the highest number of cumulative deaths, at 60 610, out of the 3 710 145 cumulative cases of COVID-19 (World Health Organization, 2022). The highest number of cases in the Philippines was documented in early January 2022, at 212 508, with a gradual decline in cases thereafter (Department of Health, 2022).

The University of the Philippines – Philippine General Hospital (UP-PGH) is a tertiary referral hospital located within the National Capital Region (NCR), which admits the most COVID-19 cases in the Philippines (Department of Health, 2022). More than 5000 patients with COVID-19 have been admitted to UP-PGH since it was designated as a COVID-19 referral center in 2020.

Early in the pandemic, when information on COVID-19 was limited, a clinical pathway for COVID-19 was created in our institution to alleviate uncertainty about COVID-19 management among healthcare workers and hospital administrators. The pathway is continuously updated as new information is published. Unfortunately, the majority of published data on COVID-19 are from middle–high-income countries, and many of the diagnostic tests and medications used are unavailable or unaffordable in low–middle-income countries (LMIC). It is therefore important to establish the experience in these LMIC countries to better tailor the approach to COVID-19 based on available resources. This is particularly relevant in our setting, as most patients hospitalized in our institution belong to the lower socioeconomic strata, pay healthcare costs out-of-pocket, and suffer loss of income and limited job opportunities as a result of the stringent COVID-19 pandemic containment measures (Ditte Fallesen, 2021).

Our study aimed to determine the predictors of mortality among adult inpatients with confirmed COVID-19 in the context of providing recommendations for resource-limited settings.

## METHODS

### Study design and setting

This was an analytic retrospective cohort study conducted at the UP-PGH. UP-PGH is a tertiary teaching COVID-19 referral center in the NCR, Philippines. The study was conducted with regulatory approval by the Institutional Review Board of UP-Manila.

### Study sample

**Figure 1:**
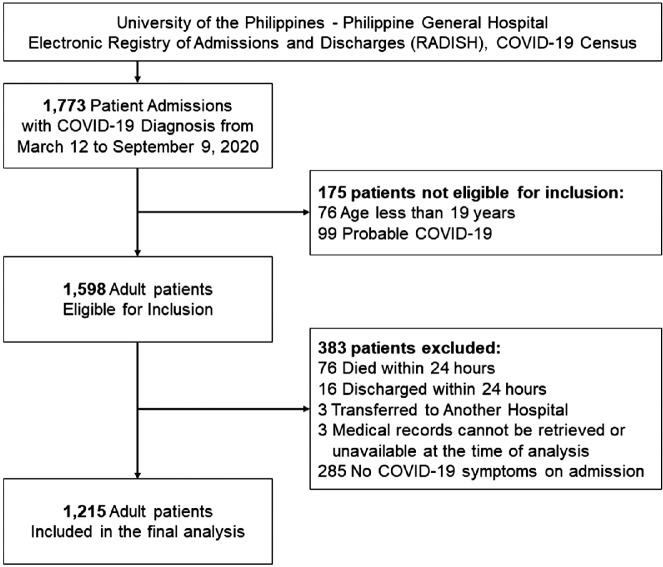
Flow diagram from the initial 1773 COVID-19 cases to the cohort of 1215 adult patients with confirmed-COVID-19 selected for the analysis of predictors of mortality

Patients diagnosed with COVID-19 infection were identified using the UP-PGH Registry of Admissions and Discharges (RADISH). Adults aged 19 years and above with confirmed COVID-19 infection were included in the study. Patients who died or were discharged within 24 hours of admission, were transferred to another hospital, whose medical records could not be retrieved, or with asymptomatic COVID-19 infection were excluded. From 1773 patients, a cohort of 1215 adult patients with confirmed COVID-19 infection, admitted between March 12 and September 9, 2021, was selected for the analysis (Figure 1).

### Data collection

Clinical and outcome data were extracted from written and electronic medical records and encoded into a Microsoft Excel worksheet. Data were extracted by a team of trained physicians from UP-PGH, while radiographic images were reviewed by the three radiologists in the team. Two study authors (AGM, JMS) reviewed the data for completeness, accuracy, and consistency. Conflicting data were resolved by consensus.

Study variables included age, sex, comorbid illnesses, symptoms, clinical findings on admission, diagnostic test results, clinical events or complications, therapeutic interventions, clinical outcome, and length of hospital stay. For specific interventions, data on antibiotic use anytime during hospitalization, and use of corticosteroids regardless of route and dose of administration, were collected.

### Definitions

A patient with *confirmed COVID-19* is anyone with a positive reverse transcription polymerase chain reaction (RT-PCR) test for severe acute respiratory syndrome coronavirus 2 (SARS-CoV-2). Illness severity was assessed on admission as follows: *mild* – symptoms consistent with COVID-19 but without evidence of pneumonia; *moderate* – symptoms consistent with COVID-19 and comorbid conditions such as hypertension, cardiovascular disease, diabetes mellitus (DM), chronic obstructive pulmonary disease (COPD), asthma, immunocompromising condition such as human immunodeficiency virus (HIV) infection, chronic steroid use, and active malignancy; or clinical and radiographic evidence of pneumonia but not requiring oxygen support; *severe* – clinical and radiographic evidence of pneumonia, with oxygen saturation ≤ 92% on room air and requiring oxygen support; and *critical* – presence of acute respiratory distress syndrome (ARDS), septic shock, requiring mechanical ventilation, or admission to the ICU.

Complications were determined using the following criteria: *acute respiratory distress syndrome* (ARDS) as per the 2012 Berlin Definitions for ARDS (ARDS Definition Task Force, 2012); *acute kidney injury* (AKI) as per the KDIGO Clinical Practice Guideline for Acute Kidney Injury (International Society of Nephrology, 2012); *acute myocardial infarction* (AMI) as per the Fourth Universal Definition of Myocardial Infarction (Thygesen et al., 2018); *pulmonary embolism* (PE) – clinical findings compatible with pulmonary embolism and documented by CT pulmonary angiogram; *acute venous thrombosis* – clinical findings compatible with deep venous thrombosis and confirmed by Duplex ultrasonography; *sepsis and septic shock* as per the Clinical Practice Guidelines for the Diagnosis and Management of Sepsis and Septic Shock in the Philippines (Clinical Practice Guidelines for Sepsis and Septic Shock Task Force, 2020). Healthcare-associated infections included hospital-acquired pneumonia (HAP) and ventilator-associated pneumonia (VAP), catheter-associated urinary tract infection (CAUTI), and catheter-related bloodstream infection (CRBSI), which were not initially present during admission. HAP and VAP were diagnosed as per the IDSA criteria (Kalil et al., 2016), CAUTI as per the Philippine Clinical Practice Guidelines for UTI in Adults criteria (Philippine CPG for UTI Task Force, 2015), and CRBSI as per the IDSA criteria (Mermel et al., 2009).

The need for supportive therapies was determined as follows: (1) *need for ICU admission* – presence of any of the following: respiratory distress requiring at least 6 lpm of oxygen support to maintain peripheral oxygen saturation (SpO_2_) > 92%; rapid escalation of oxygen requirements or significant work of breathing; hemodynamic instability with systolic blood pressure (SBP) < 90 mmHg, mean arterial pressure (MAP) < 65, or heart rate (HR) > 120 beats/minute; acidosis with arterial blood pH < 7.3 or pCO_2_ > 50, and/or lactate > 2; or any physician concern or need for closer monitoring in the ICU; and (2) *need for renal replacement therapy* (RRT) – occurrence of any indications for renal replacement therapy, such as uremia, refractory acidosis, severe hyperkalemia or hypercalcemia, oliguria/anuria, or volume overload unresponsive to diuretic therapy.

In-hospital mortality was defined as death from any cause during the hospital stay. Survivors included patients who remained alive until hospital discharge, while non-survivors included those who died during the hospital stay

### Statistical analysis

Descriptive statistics were used and frequency distributions of demographic and clinical characteristics determined. The Shapiro-Wilk test was used to assess the normality of continuous data, and values were expressed as median and interquartile range (IQR). Univariate analyses using chi-square for categorial variables and the Mann-Whitney test for continuous variables were performed to compare the clinical characteristics of survivors and non-survivors on hospital admission.

Multivariate analyses were performed to determine the predictors of in-hospital mortality in our cohort, using variables obtained on admission. Variables commonly associated with mortality were selected, based on published data (Izcovich et al., 2020; Mesas et al., 2020). Variables with more than 15% missing data, namely procalcitonin and D-dimer, were excluded. All 20 variables selected were assessed for missingness, with the proportion missing for each variable outlined in Supplementary Table 1. Missingness was assumed to be missing at random (MAR), with missing variables imputed using multiple imputation by chained equations (MICE) to allow for flexibility, given that the predictors were a mix of continuous and dichotomous measures. In total, 15 imputations with 10 iterations each were created. The imputation model included the following covariates due to biological correlations with one or more of the 20 variables of interest: illness severity on admission, creatinine, hemoglobin, SpO_2_, cancer, and chronic liver disease (CLD). Imputation was performed using Stata/IC 15.1. No interaction terms were assumed or included in the imputation model. The multiply imputed data sets were then dichotomized for clinical interpretability and then analyzed using a multiple logistic regression model. The magnitude of association was expressed as odds ratio (OR) with 95% confidence interval (CI). For laboratory parameters found to be associated with mortality, mean values between survivors and non-survivors were plotted and compared throughout the first 4 weeks of illness from symptom onset. *Post hoc* analysis that included tuberculosis in the multivariable regression was also performed.

Frequencies of clinical events and complications observed in the cohort were determined; these included need for oxygen support, need for invasive ventilation, need for ICU admission, ARDS, AKI, need for RRT, acute stroke, AMI, PE, DVT, sepsis, septic shock, HAI, nosocomial pneumonia, CAUTI, and CRBSI. The risks of death associated with these events were analyzed using chi-square, and the magnitude of association expressed as OR with 95% CI.

All tests were two-tailed, with *p*-values less than 0.05 considered statistically significant. Analyses were conducted using Stata/IC 15.1 and MedCalc.

## RESULTS

### Characteristics of the study cohort

The patients’ demographic and clinical characteristics, and the therapeutic interventions they received, are listed in Table 1. A greater proportion of patients who died were ≥ 60 years, male, had pre-existing comorbid illness (e.g. hypertension, CLD, COPD, asthma, active pulmonary tuberculosis, cancer, or neurological disease), and had history of smoking and alcohol consumption (*p* < 0.05). More patients who did not survive also presented with shortness of breath, decreased appetite, and changes in sensorium (*p* < 0.05). On hospital admission, non-survivors had higher median heart rate, respiratory rate, and temperature, while peripheral oxygen saturation and Glasgow coma scale (GCS) scores were lower than among the survivors (*p* < 0.05).

**Table 1:** Demographics, clinical characteristics on admission, and therapeutic interventions received by COVID-19 patients in the cohort.

	CLINICAL OUTCOME			
	OVERALL	SURVIVOR	NONSURVIVOR	P value
	(N=1215)	(N=994)	(N=221)	
**AGE**				
Median, IQR	55 (42 to 66)	52 (38 to 63)	65 (56 to 75)	<0.01
60 years and above, No. (%)	473 (38.9)	329 (33.1)	144 (65.2)	<0.01
**SEX**, No. (%)				
Male	638 (52.5)	504 (50.7)	134 (60.6)	<0.01
**COEXISTING CONDITION**, No. (%)				
Presence of any comorbid illness	875 (72.0)	686 (69.0)	189 (85.5)	<0.01
Diabetes mellitus	311 (25.6)	245 (24.6)	66 (29.9)	0.11
Hypertension	583 (48.0)	457 (46.0)	126 (57.0)	<0.01
Heart disease	165 (13.6)	126 (12.7)	39 (17.6)	0.05
Chronic liver disease	11 (0.9)	7 (0.7)	4 (1.8)	0.01
Chronic kidney disease	108 (8.9)	82 (8.2)	26 (11.8)	0.10
COPD	29 (2.4)	17 (1.7)	12 (5.4)	<0.01
Asthma	87 (7.2)	78 (7.8)	9 (4.1)	0.05
Active pulmonary tuberculosis	39 (3.2)	25 (2.5)	14 (6.3)	<0.01
HIV	7 (0.6)	5 (0.5)	2 (0.9)	0.47
Cancer	70 (5.8)	47 (4.7)	23 (10.4)	<0.01
Neurologic disease	84 (6.9)	55 (5.5)	29 (13.1)	<0.01
Smoker	258 (21.2)	187 (18.8)	71 (32.1)	<0.01
Alcohol beverage drinker	289 (23.8)	223 (22.4)	66 (29.9)	0.03
History of Illicit drug use	22 (1.8)	16 (1.6)	6 (2.7)	0.29
**SPECIAL POPULATION**				
Healthcare workers	257 (21.2)	254 (25.6)	3 (1.4)	<0.01
Pregnant	24 (2.0)	23 (2.3)	1 (0.5)	0.07
**SYMPTOMS**, No (%)				
Headache	101 (8.3)	93 (9.4)	8 (3.6)	<0.01
Chills	51 (4.2)	45 (4.5)	6 (2.7)	0.22
Fever	703 (57.9)	577 (58.0)	126 (57.0)	0.78
Cough	752 (61.9)	604 (60.8)	148 (67.0)	0.09
Rhinorrhea / Congestion	163 (13.4)	150 (15.1)	13 (5.9)	<0.01
Shortness of Breath / Dyspnea	558 (45.9)	406 (40.8)	152 (68.8)	<0.01
Sore throat	183 (15.1)	164 (16.5)	19 (8.6)	<0.01
Myalgia	94 (7.7)	87 (8.8)	7 (3.2)	<0.01
Malaise / Fatigue /				
Generalized Weakness	345 (28.4)	273 (27.5)	72 (32.6)	0.13
Diarrhea	199 (16.4)	171 (17.2)	28 (12.7)	0.10
Nausea or Vomiting	65 (5.3)	48 (4.8)	17 (7.7)	0.09
Decreased Appetite	161 (13.3)	114 (11.5)	47 (21.3)	<0.01
Abdominal pain / discomfort	58 (4.8)	45 (4.5)	13 (5.9)	0.39
Change or Loss in Taste	94 (7.7)	80 (8.0)	14 (6.3)	0.39
Change or Loss in Smell	94 (7.7)	89 (9.0)	5 (2.3)	<0.01
Decreased Sensorium	88 (7.2)	42 (4.2)	46 (20.8)	<0.01
**VITAL SIGNS ON ADMISSION**, median (IQR)				
Systolic blood pressure, mmHg	130 (117 to 140)	128 (117 to 140)	130 (118 to 150)	0.01
Diastolic blood pressure, mmHg	80 (70 to 85)	80 (70 to 85)	79 (70 to 87)	0.04
Mean arterial pressure, mmHg	93 (87 to 103)	93 (87 to 103)	97 (87 to 104)	0.50
Heart rate, beats/min	87 (79 to 100)	86 (78 to 96)	101 (85 to 114)	<0.01
Respiratory rate, breaths/min	20 (20 to 24)	20 (20 to 22)	26 (23 to 30)	<0.01
Temperature, degrees Celsius	36.6 (36.3 to 37.0)	36.5 (36.2 to 37.0)	36.8 (36.5 to 37.2)	<0.01
Peripheral oxygen saturation, %	97 (94 to 98)	97 (95 to 98)	92 (80 to 97)	<0.01
Glasgow Coma Scale Score	15 (15 to 15)	15 (15 to 15)	15 (13 to 15)	<0.01
**LABORATORY FINDINGS**				
Complete Blood Count, median (IQR)				
Hemoglobin	132 (116 to 144)	133 (120 to 145)	121 (98 to 140)	<0.01
Hematocrit	40 (35 to 43)	40 (36 to 43)	37 (30 to 43)	<0.01
White blood cell count	7.7 (5.7 to 10.7)	7.4 (5.6 to 9.6)	11.3 (7.6 to 15.5)	<0.01
Neutrophil percentage	70.0 (59.0 to 82.0)	67.0 (57.0 to 77.0)	85.0 (77.8 to 90.0)	<0.01
Lymphocyte percentage	18.0 (10.0 to 28.0)	21.0 (13.0 to 30.0)	7.0 (5.0 to 13.3)	<0.01
Absolute lymphocyte count	1350 (874 to 1924)	1463 (996 to 2000)	848 (561 to 1277)	<0.01
Neutrophil lymphocyte ratio	3.8 (2.1 to 8.3)	3.2 (1.9 to 6.0)	12.0 (5.8 to 18.4)	<0.01
Platelet count	272 (202 to 354)	282 (208 to 361)	250 (171 to 331)	<0.01
Arterial blood gas, median (IQR)				
pH	7.42 (7.39 to 7.46)	7.43 (7.40 to 7.46)	7.40 (7.3 to 7.5)	<0.01
pCO_2_	34.9 (29.2 to 39.0)	35.0 (30.1 to 39.0)	31.9 (27.0 to 37.0)	<0.01
pAO_2_	90.0 (75.3 to 106.9)	90.4 (78.0 to 106.0)	83.0 (63.2 to 117.8)	0.01
HCO_3_	22.9 (19.0 to 25.7)	23.5 (20.0 to 25.9)	18.8 (16.1 to 22.7)	<0.01
O_2_ saturation	97.0 (95.0 to 98.0)	97.0 (96.0 to 98.0)	96.0 (91.3 to 98.4)	<0.01
PaO_2_ and FiO_2_ ratio	376 (240 to 454)	395 (295 to 462)	176 (107 to 342)	<0.01
Blood Chemistry, median (IQR)				
BUN, mmol/L	5.1 (3.7 to 8.9)	4.7 (3.5 to 7.0)	11.0 (6.0 to 21.8)	<0.01
Serum creatinine, µmol/L	75.0 (57.0 to 113.0)	71.0 (56.0 to 94.0)	115.0 (75.8 to 276.3)	<0.01
eGFR*, mL/min/1.73m^2^	91.0 (54.0 to 109.0)	95.0 (68.0 to 112.0)	51.0 (18.8 to 83.3)	<0.01
AST, U/L	47.0 (32.0 to 75.0)	43.0 (31.0 to 67.0)	64.0 (47.0 to 98.0)	<0.01
ALT, IU/L	38.0 (21.0 to 70.0)	39.0 (21.0 to 70.8)	35.5 (21.0 to 69.0)	0.94
Albumin, g/L	37.0 (32.0 to 42.0)	38.0 (34.0 to 43.0)	33.0 (29.0 to 37.0)	<0.01
Total bilirubin, mg/dl	0.68 (0.50 to 0.990	0.65 (0.49 to 0.94)	0.89 (0.57 to 1.34)	<0.01
Direct bilirubin, mg/dl	0.29 (0.20 to 0.45)	0.26 (0.18 to 0.38)	0.45 (0.33 to 0.75)	<0.01
Indirect bilirubin, mg/dl	0.38 (0.22 to 0.60)	0.37 (0.23 to 0.58)	0.42 (0.18 to 0.69)	0.55
Inflammatory Markers, median (IQR)				
LDH, U/L	318 (240 to 479)	297 (230 to 413)	547 (360 to 827)	<0.01
Serum ferritin, ng/mL	588 (209 to 1320)	473 (179 to 1095)	1320 (730 to 2760)	<0.01
Serum procalcitonin, ng/mL	0.16 (0.04 to 0.62)	0.09 (0.04 to 0.35)	0.97 (0.29 to 3.60)	<0.01
D-dimer, ug/mL	1.34 (0.60 to 3.16)	0.98 (0.48 to 2.28)	3.39 (1.65 to 9.28)	<0.01
C-reactive protein, No. (%)				
No CRP determination	161 (13.3)	136 (13.7)	25 (11.3)	
≤12 mg/L	402 (33.1)	387 (38.9)	15 (6.8)	
>12 mg/L	652 (53.7)	471 (47.4)	181 (81.9)	<0.01
**CHEST RADIOGRAPH**, No. (%)				
No Chest X-ray	7 (0.6)	5 (0.5)	2 (0.9)	
Pulmonary Infiltrates				
Bilateral	689 (56.7)	507 (51.0)	182 (82.4)	<0.01
More than 50% of the lungs	484 (39.8)	329 (33.1)	155 (70.1)	<0.01
Limited to periphery	84 (6.9)	75 (7.5)	9 (4.1)	0.07
Density				
Ground Glass	615 (50.6)	456 (45.9)	159 (71.9)	<0.01
Consolidation	103 (8.5)	62 (6.2)	41 (18.6)	<0.01
Other Findings				
Pleural effusion	92 (7.6)	64 (6.4)	28 (12.7)	<0.01
Pneumothorax	5 (0.4)	3 (0.3)	2 (0.9)	0.20
**ILLNESS SEVERITY ON ADMISSION**, No. (%)				
Mild	203 (16.7)	203 (20.4)	0 (0)	
Moderate	488 (40.2)	458 (46.1)	30 (13.6)	<0.01
Severe	183 (15.1)	154 (15.5)	29 (13.1)	<0.01
Critical	341 (28.1)	179 (18.0)	162 (73.3)	<0.01
**STATUS ON ADMISSION**, No (%)				
Requiring oxygen support	521 (42.9)	330 (33.2)	191 (86.4)	<0.01
On ventilatory support	97 (8.0)	16 (1.6)	81 (36.7)	<0.01
Acute respiratory distress syndrome	250 (20.6)	146 (14.7)	104 (47.1)	<0.01
On vasopressor	32 (2.6)	4 (0.4)	28 (12.7)	<0.01
**INTERVENTIONS**				
Antibiotics	802 (66.0)	581 (58.5)	221 (100)	<0.01
Corticosteroids	443 (36.5)	289 (29.1)	154 (69.7)	<0.01
Remdesivir	115 (9.5)	91 (9.2)	24 (10.9)	0.43
Interferon beta 1a	20 (1.6)	18 (1.8)	2 (0.9)	0.34
Tocilizumab	176 (14.5)	94 (9.5)	82 (37.1)	<0.01
Hydroxychloroquine	90 (7.4)	70 (7.0)	20 (9.0)	0.30
Lopinavir/ritonavir	32 (2.6)	23 (2.3)	9 (4.1)	0.14
Convalescent Plasma	49 (4.0)	24 (2.4)	25 (11.3)	<0.01
Hemoperfusion	74 (6.1)	32 (3.2)	42 (19.0)	<0.01

ALT – alanine aminotransferase; AST – aspartate aminotransferase; COPD – Chronic obstructive pulmonary disease; eGFR – estimated glomerular filtration rate; HIV – Human immunodeficiency virus; LDH – lactate dehydrogenase;

*The CKD-EPI (Chronic Kidney Disease Epidemiology Collaboration) equation was used to estimate GFR.

Laboratory tests requested on admission included complete blood count (CBC; 96.5%, *n* = 1173), arterial blood gas (ABG; 92.4%, *n* = 1123), blood urea nitrogen (BUN; 91.7%, *n* = 1114), serum creatinine (94.7%, *n* = 1151), aspartate aminotransferase (AST; 90.8%, *n* = 1103), alanine aminotransferase (ALT; 91.3%, *n* = 1109), lactate dehydrogenase (LDH; 91.9%, *n* = 1116), serum ferritin (93.2%, *n* = 1132), albumin (78.9%, *n* = 959), bilirubins (75.1%, *n* = 913), and C-reactive protein (CRP; 86.7%, *n* = 1054). Procalcitonin and D-dimer levels were measured in 54.7% (*n* = 664) and 64.0% (*n* = 777) of patients, respectively. Comparisons of medians and IQR values for various laboratory parameters between survivors and non-survivors are summarized in Table 1. About 99.4% (*n* = 1208) of the patients had a chest radiograph taken on admission. Patients who died had more lung abnormalities and extensive lung involvement compared with those who survived (Table 1).

Compared with survivors, a large proportion of patients who died had evidence of critical COVID-19 on admission (18.0% vs 73.3%), and were more likely to require oxygen support (33.2% vs 86.4%), invasive ventilatory support (1.6% vs 36.7%), and vasopressor support (0.4% vs 12.7%). Nearly half (47.1%) of patients who died had ARDS on admission. More non-survivors received antibiotics, corticosteroids, tocilizumab, and convalescent plasma, and underwent hemoperfusion (*p* < 0.05, Table 1). Of the 443 patients given corticosteroids, 393 (88.7% overall; 263/289, 91.0% survivors; 130/154, 84.4% non-survivors) received a dose similar to that used in the trial by the Recovery Collaborative Group (RECOVERY Collaborative Group, 2021).

### Factors associated with in-hospital mortality

The in-hospital mortality rate for symptomatic patients with COVID-19 was 18.2% (*n* = 221). Rates by illness severity were 0% (0/203) for mild, 6.1% (30/488) for moderate, 15.8% (29/183) for severe, and 47.5% (162/341) for critical cases. The most common cause of death was acute respiratory failure or ARDS from COVID-19 (42.9%, *n* = 97), followed by septic shock from nosocomial pneumonia (14.2%, *n* = 32), and acute coronary syndrome (11.5%, *n* = 26).

Predictors of in-hospital mortality in our cohort were age ≥ 60 years, COPD, qSOFA score ≥ 2, leukocytosis (WBC > 10 × 10^9^/L), lymphopenia (ALC < 1000), neutrophilia (neutrophil ≥ 70%), PaO_2_/FiO_2_ ratio (PFR) ≤ 200, estimated glomerular filtration rate (eGFR) < 90 mL/min/1.73 m^2^, LDH > 600 U/L, and CRP > 12 mg/L. Table 2 shows the variables included in the model and the corresponding odds of mortality (95% CI). *Post hoc* analysis, which incorporated active tuberculosis in the model, exhibited a trend towards increased mortality, though this was not statistically significant (Supplementary Table 2).

**Table 2:** Multivariate analysis of predictors of mortality among hospitalized COVID-19 patients.

Parameters	Odds ratio	(95% Confidence Interval)	P value
Reference	0.01	(0.004` - 0.03)	
Age ≥ 60 years	1.93	(1.25 - 2.98)	<0.01
Male	0.76	(0.49 - 1.18)	0.22
Hypertension	0.71	(0.47 - 1.09)	0.12
Diabetes mellitus	1.07	(0.69 - 1.67)	0.76
Heart Disease	1.10	(0.64 - 1.89)	0.74
Chronic Obstructive Pulmonary Diseases	2.68	(1.01 - 7.14)	0.05
Chronic Kidney Disease	1.07	(0.57 - 1.99)	0.84
Neurologic Disease	1.42	(0.73 - 2.77)	0.30
Smoker	1.38	(0.85 - 2.25)	0.19
Shortness of breath	1.52	(1.00 - 2.30)	0.05
qSOFA score ≥ 2	7.95	(4.58 - 13.78)	<0.01
White Blood Cell Count			
< 4 x 10^9^/L	0.37	(0.10 - 1.39)	0.14
4 to 10 x 10^9^/L	Ref		
> 10 x 10^9^/L	1.58	(1.02 - 2.45)	0.04
Absolute lymphocyte count < 1000	1.83	(1.18 - 2.84)	<0.01
Percent Neutrophil ≥ 70	2.45	(1.31 - 4.58)	<0.01
Platelet count			
< 100 x 10^9^/L	1.01	(0.33 - 3.10)	0.99
100 to 150 x 10^9^/L	Ref		
> 150 x 10^9^/L	0.44	(0.22 - 0.89)	0.02
PaO_2_/FiO_2_ Ratio			
≤100	2.79	(1.42 - 5.48)	<0.01
101-200	1.99	(1.15 - 3.44)	0.01
201-300	1.01	(0.56 - 1.82)	0.97
>300	Ref		
eGFR < 90 mL/min/1.73m^2^	1.78	(1.10 - 2.88)	0.02
Lactate dehydrogenase ≥ 600 U/L	2.28	(1.41 - 3.69)	<0.01
Serum ferritin ≥ 600	1.25	(0.74 - 2.10)	0.40
C-reactive protein ≥ 12 mg/L	3.15	(1.53 - 6.50)	<0.01

eGFR – estimated glomerular filtration rate; qSOFA – quick sequential organ failure assessment

*The CKD-EPI (Chronic Kidney Disease Epidemiology Collaboration) equation was used to estimate GFR

**Figure 2:**
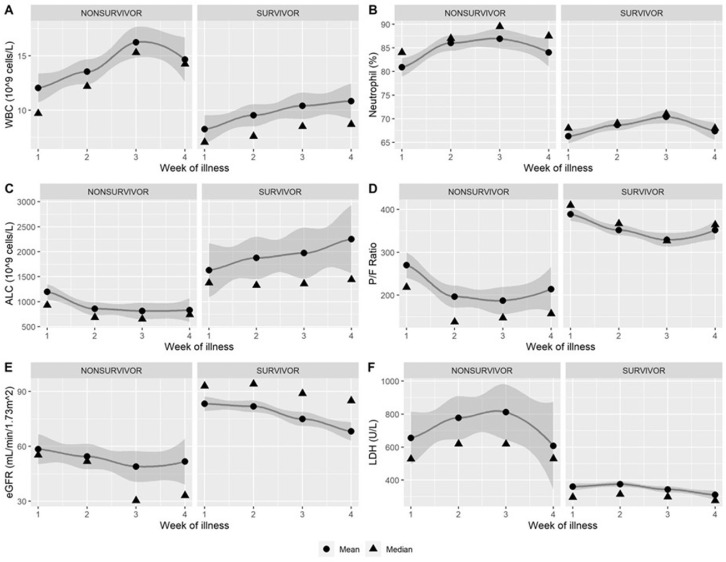
Comparisons between COVID-19 survivors and nonsurvivors for the different laboratory findings throughout the course of illness. Dots represent mean values, while triangles represent median values. Gray areas correspond to the standard error. A. white blood cell count; B. neutrophil percentage; C. absolute lymphocyte count (ALC); D. PaO_2_/FiO_2_ ratio; E. estimated glomerular filtration rate (eGFR) calculated using the CKD-EPI (Chronic Kidney Disease Epidemiology Collaboration) equation; F. lactate dehydrogenase (LDH).

Figure 2 shows the trends in laboratory parameters throughout the course of illness for survivors and non-survivors. Compared with survivors, non-survivors exhibited persistent leukocytosis, neutrophilia, hypoxemia, and elevation in LDH throughout the 4-week period from the onset of symptoms. Furthermore, lymphopenia and further decline in pulmonary function (by PFR) and renal function (by eGFR) among non-survivors occurred during the 2nd and 3rd weeks of illness.

### Other clinical outcomes

The frequencies of various clinical events and complications among patients with COVID-19 are shown in Table 3. The odds of dying were highest among patients who required invasive ventilation (OR 481.00; 95% CI 248.15–932.34), followed by those who developed septic shock (OR 113.04; 95% CI 66.15–193.17), required ICU admission (OR 92.96; 95% CI 43.19–200.06), and required oxygen support (OR 89.65; 95% CI 28.49–282.06). Patients who developed healthcare-associated infections, particularly nosocomial pneumonia, were at a higher risk of mortality compared with those with secondary infections from non-pulmonary etiologies.

**Table 3:** Mortality risk among COVID-19 patients admitted in UP-PGH who developed complications and on supportive therapies

		INHOSPITAL OUTCOME			
Overall	Survivor	Nonsurvivor	OR	95% Confidence Interval	P value
	(N=1215)	(N=994)	(N=221)		
**SUPPORTIVE THERAPY**					
Need for oxygen support	663 (54.6)	445 (44.8)	218 (98.6)	89.65 (28.49 to 282.06)	<0.01
Need for invasive ventilation	240 (19.8)	32 (3.2)	208 (94.1)	481.00 (248.15 to 932.34)	<0.01
Need for ICU admission	460 (37.9)	246 (24.7)	214 (96.8)	92.96 (43.19 to 200.06)	<0.01
Need for renal replacement therapy	113 (9.3)	42 (4.2)	71 (32.1)	10.73 (7.06 to 16.31)	<0.01
**COMPLICATIONS**					
Acute respiratory distress syndrome	380 (31.3)	206 (20.7)	174 (78.7)	14.16 (9.91 to 20.23)	<0.01
Acute kidney injury	217 (17.9)	90 (9.1)	127 (57.5)	13.57 (9.62 to 19.13)	<0.01
Acute stroke	47 (3.9)	20 (2.0)	27 (12.2)	6.78 (3.73 to 12.33)	<0.01
Acute myocardial infarction	44 (3.6)	13 (1.3)	31 (14.0)	12.31 (6.33 to 23.97)	<0.01
Deep venous thrombosis or Pulmonary embolism	19 (1.6)	7 (0.7)	12 (5.4)	8.10 (3.15 to 2.81)	<0.01
Sepsis	329 (27.1)	139 (14.0)	190 (86.0)	37.70 (24.77 to 57.37)	<0.01
Septic shock	171 (14.1)	19 (1.9)	152 (68.8)	113.04 (66.15 to 193.17)	<0.01
Healthcare-associated infection*	208 (17.1)	105 (10.6)	103 (46.6)	7.39 (5.30 to 10.31)	<0.01
Nosocomial pneumonia	198 (16.3)	97 (9.8)	101 (45.7)	7.78 (5.55 to 10.91)	<0.01
Catheter-associated urinary tract infection	9 (0.7)	5 (0.5)	4 (1.8)	3.65 (0.97 to 13.69)	0.05
Catheter-related bloodstream infection	13 (1.1)	8 (0.8)	5 (2.3)	2.85 (0.92 to 8.81)	0.07

* One patient may have multiple sites of infection identified

The median duration of hospitalization was 13 days (IQR 8–20). Duration of hospitalization was significantly shorter for non-survivors compared with survivors, with a median of 7 days (IQR 4–14) versus 13 days (IQR 9–21) (*p* < 0.001), respectively.

## DISCUSSION

Our report provides important epidemiological data from a large cohort of confirmed COVID-19 patients in the Philippines, an LMIC, before the emergence of SARS-CoV-2 variants. Our reported in-hospital mortality rate of 18.2% was comparable with those in local studies conducted during the same period. A 200-patient cohort from the same institution reported a 17.5% mortality rate (Salamat et al., 2021), while a nationwide multicenter study that included 10 881 patients reported a 15.6% mortality rate (Espiritu et al., 2021). A government and a private tertiary hospital in NCR reported mortality rates that closely approximated our data at 21% and 15%, respectively (Abad et al., 2021; Salva et al., 2020).

During the same period, in-hospital mortality rates abroad were slightly higher, ranging from 21.7% to 29.7% (Bellan et al., 2020; Mikami et al., 2021; Zhou et al., 2020). This was attributed to the large proportion of patients with severe disease. In contrast, the in-hospital mortality rate in South Korea was low, at 1.1%, because the majority (91%) had mild disease (Sung et al., 2020). In our cohort, nearly half (43.1%) presented with severe-to-critical disease. The differences in mortality among regions may be explained by the underlying health infrastructures and policies in place. For example, the Philippines implemented the longest and strictest lockdown in the world (Aie Balagtas See, 2021) which could have mitigated the rise in cases. However, other factors could have influenced the mortality rates, such as poor healthcare-seeking behavior, undertesting, underreporting, and limited access to COVID-19 services (Bajo, 2022).

The result of the multivariate analysis of the predictors of mortality supported the findings of systematic reviews and meta-analyses (Katzenschlager et al., 2021; Shi et al., 2021). Age has always been identified as an independent predictor of mortality, with immunosenescence, age-related physiological changes, and preexisting illnesses cited as reasons for increased vulnerability (Shi et al., 2021). Individuals with COPD have an inherent pulmonary risk because of poor lung function and immune modulation of the airways. A population-based study in South Korea showed an independent association of COPD with mortality (Lee et al., 2021). However, our study found no association with other commonly cited predictors of mortality – male sex, smoking, DM, CKD, cerebrovascular disease, and cardiovascular disease. Although beyond the scope of our study, it is possible that the patients in our cohort had comorbid illnesses that were either newly diagnosed or well controlled. Other studies have reported that the level of control and the presence of complications are determinants of increased mortality. In England, for example, hyperglycemia, HbA_1c_ > 7.6%, obesity, and the presence of cardiovascular and renal complications were found to be independently associated with mortality among diabetics who had COVID-19 infection (Holman et al., 2020). Unfortunately, in our study, data required for calculating body mass index (BMI) or assessing DM control could not be obtained.

Renal status was estimated by calculating eGFR using serum creatinine levels obtained on admission. An eGFR < 90 mL/min/1.73 m^2^ indicated renal dysfunction; however, whether this was acute or chronic could not be determined in all cases. Nevertheless, studies have shown increased mortality risk among those with acute renal complications, as well as those with CKD (Alenezi et al., 2021; Mohamed et al., 2021; Pecly et al., 2021). In our study, mortality was predicted by an eGFR < 90 mL/min/1.73 m^2^ but not by CKD. It is possible that some of those with an eGFR < 90 mL/min/1.73 m^2^ may have had undiagnosed CKD; the majority of patients admitted to our institution are from the marginalized sector, and are less likely to seek medical consultation. This can result in the underreporting of CKD.

Our mortality estimates were adjusted to consider age, which possibly explains the lack of association detected among those with cerebro- and cardiovascular diseases. These diseases are more prevalent among the elderly and are usually complications of an underlying condition (hypertension and DM). For men, genetic and hormonal predisposition are still being explored, but the higher prevalence of cardiovascular comorbidities in this group could have contributed to the increased mortality observed in other studies (Bienvenu et al., 2020; Penna et al., 2020). For smokers, our data may have suggested a lower rate than reality due to possible underreporting, i.e. physicians not completing all information on the clinical pathway form.

Non-survivors exhibited lymphopenia, which became evident during the second week of illness. A significant reduction in lymphocyte count has been reported as a marker of severe disease and in-hospital mortality in other systematic reviews and meta-analyses (Henry et al., 2020; Malik et al., 2021). Lymphocyte counts of < 1500/μL carry a threefold higher risk of poor outcomes (pooled OR 3.47; 95% CI 2.77–4.36; *p* < 0.01) (Malik et al., 2021). In COVID-19, both effector and memory lymphocytes are greatly diminished, with the latter potentially resulting in poor immunity against future infection (Delshad et al., 2021). Proposed mechanisms leading to lymphopenia include cytokine storm, which upregulates substances that induce T cell apoptosis, direct infection of lymphatic organs, with atrophy and destruction of germinal centers, bone marrow suppression, lactic acidemia, causing inhibition of lymphocyte proliferation, and alteration in gene expression, which affects lymphocyte proliferation and activity (Delshad et al., 2021).

Non-survivors also showed signs of marked inflammation, manifesting as leukocytosis, neutrophilia, and elevated LDH or CRP. Some exhibited signs of organ dysfunction (decreased eGFR, hypoxemia) and sepsis (qSOFA > 2). Intense inflammation can drive acute lung injury and ARDS, and can also lead to multiple organ failure (Hu et al., 2021). The odds of dying were more than 10 times higher for patients who developed sepsis and septic shock, ARDS, AKI, and AMI (Table 3). This was also observed among the patients who required ICU care, oxygen therapy, invasive mechanical ventilation, and RRT. Our data reflected findings in the current literature – that development of ARDS, need for invasive ventilation, ICU admission, and RRT are associated with higher mortality (Potere et al., 2020).

## Clinical implications

With the continuing threat of SARS-CoV-2 and its variants, there is a need to maximize the use of clinical pathways to efficiently respond to the surge in cases, especially in resource-poor settings. Several clinical calculators have been designed to estimate mortality risk (Garibaldi et al., 2021; Jin et al., 2021; QxMD Software Inc., 2020), but these are often difficult to apply to our setting and do not provide definite guidance on the subsequent steps. In addition, the required diagnostic tests are either expensive or unavailable in resource-poor areas. Our study found the following common and inexpensive laboratory tests to be essential in the initial evaluation of a patient with COVID-19: CBC, ABG, serum creatinine, LDH, CRP, and chest X-ray. Calculations of qSOFA scores and eGFRs are recommended to assess baseline risk and the need for further intervention and monitoring.

In terms of monitoring, our results suggest that repeat measurements should be deferred until the second week if initial test results during the first week of illness are low or within normal limits. Those who have abnormal test results upon admission, and those who exhibit signs of clinical worsening (e.g. progressive dyspnea) may need more frequent monitoring, especially during the 2nd and 3rd weeks of illness, when there are signs of hypoxemia (PFR ≤ 200), increased inflammation (LDH ≥ 600, increased CRP), and declining renal status (eGFR < 90). The results of these tests can guide pharmacological management, oxygen therapy, and initiation of other supportive strategies. Serial measurements can be discontinued once parameters show signs of improvement.

## Study limitations

Our data were limited to the period when the wild-type SARS-CoV-2 was predominant, hence the mortality data may not be comparable to current data. Recent reports of COVID-19 outbreaks involving the variants of concern (VOC) reveal higher mortality than those involving the wild-type SARS-CoV-2 (Challen et al., 2021; Venkatraja et al., 2022).

The study was also limited by its retrospective nature, with some missing or incomplete information. Nonetheless, information bias was minimized by the COVID-19 clinical pathway being implemented in our institution.

Data on glycemic control among diabetics and BMI were not obtained. Moreover, nearly half of the patients did not undergo procalcitonin and D-dimer determination. Thus, the clinical relevance of these variables could not be evaluated.

Finally, the effect of interventions was not included in the multivariate analysis. Our study covered the early period of the pandemic, when very limited therapeutic options for COVID-19 were available. The earliest evidence on the benefit of corticosteroids emerged in July 2020 (RECOVERY Collaborative Group, 2021), which was midway through the study period. Nevertheless, a greater proportion of survivors vs non-survivors (91.0% vs 84.4%) received corticosteroids.

## CONCLUSIONS

In-hospital mortality in our institution was comparable to local data early in the pandemic, when only the wild-type SARS-CoV-2 strains were circulating. Predictors of in-hospital mortality were similar to global reports, except that, among comorbidities, the only association was found to be with COPD. Marked inflammation and worsening pulmonary and renal function were observed among non-survivors by the 2nd–3rd week of illness, which may indicate the critical period when closer monitoring is necessary. The odds of dying were found to be higher for those with complications and those who required oxygen support, invasive ventilation, ICU admission, and renal replacement therapy.

## FUNDING

This research did not receive any specific grant from funding agencies in the public, commercial, or not-for-profit sectors.

## AUTHOR CONTRIBUTIONS

Conceptualization/investigation – all authors; data collection – AGM, JMS, JBP, JGP, SLM, RWG, AME, JAC, JAS, JTM; data validation – AGM, JMS; data analysis – AGM, CRA, MSS, MMA, MPM; resources/software – AGM, MPM; supervision – MMA, MSS, CRA, JAC; writing, original draft – AGM, JBP, RWG, RDR, EBO; writing, review and editing – AGM, CRA, MSS, MMA.

## ETHICAL APPROVAL

The study was conducted with regulatory approval by the Institutional Review Board of UP-Manila.

## Declaration of Competing Interest

The authors declare no conflicts of interest.

## Appendix Supplementary materials

Image, application 1
