# Supplementary material for: Predictors of mortality among inpatients with COVID-19 infection in a tertiary referral center in the Philippines
Source: IJID Reg. 2022 Jul 14;4:134–42. doi: 10.1016/j.ijregi.2022.07.009 (PMC9281405; doi:10.1016/j.ijregi.2022.07.009)
Supplement: Supplementary file 1 [file mmc1.docx]

Supplementary Table 1. Multivariable Logistic Regression on the Predictors of Mortality in COVID-19

**Parameters Percent Multiple Imputation by chained equations**

**Missing OR (95% CI) P value**

Reference 0.01 (0.004 - 0.03)

Age ≥ 60 years 0 1.93 (1.25 - 2.98) <0.01

Male 0 0.76 (0.49 - 1.18) 0.22

Hypertension 0 0.71 (0.47 - 1.09) 0.12

Diabetes mellitus 0 1.07 (0.69 - 1.67) 0.76

Heart Disease 0 1.10 (0.64 - 1.89) 0.74

COPD 0 2.68 (1.01 - 7.14) <0.05

Chronic Kidney Disease 0 1.07 (0.57 - 1.99) 0.84

Neurologic Disease 0 1.42 (0.73 - 2.77) 0.30

Smoker 4.9 1.38 (0.85 - 2.25) 0.19

Shortness of breath 0 1.52 (1.00 - 2.30) 0.05

qSOFA score ≥ 2 0 7.95 (4.58 - 13.78) <0.01

White Blood Cell Count 3.5

< 4 x 10^9^/L 0.37 (0.10 - 1.39) 0.14

4 to 10 x 10^9^/L Ref

> 10 x 10^9^/L 1.58 (1.02 - 2.45) 0.04

Absolute lymphocyte count < 1000 3.6 1.83 (1.18 - 2.84) <0.01

Percent Neutrophil ≥ 70 3.6 2.45 (1.31 - 4.58) <0.01

Platelet count 3.8

< 100 x 10^9^/L 1.01 (0.33 - 3.10) 0.99

100 to 150 x 10^9^/L Ref

> 150 x 10^9^/L 0.44 (0.22 - 0.89) 0.02

PaO_2_/FiO_2_ Ratio 7.6

≤100 2.79 (1.42 - 5.48) <0.01

101-200 1.99 (1.15 - 3.44) 0.01

201-300 1.01 (0.56 - 1.82) 0.97

>300 Ref

eGFR < 90 5.3 1.78 (1.10 - 2.88) 0.02

Lactate dehydrogenase ≥ 600 U/L 8.2 2.28 (1.41 - 3.69) <0.01

Serum ferritin ≥ 600 5.3 1.25 (0.74 - 2.10) 0.40

C-reactive protein ≥ 12 mg/L 13.0 3.15 (1.53 - 6.50) <0.01

COPD – Chronic obstructive pulmonary disease; eGFR – estimated glomerular filtration rate; qSOFA – quick sequential organ failure assessment

*The CKD-EPI (Chronic Kidney Disease Epidemiology Collaboration) equation was used to estimate GFR

Supplementary Table 2. *Post-hoc Analysis including tuberculosis*, Multivariable Logistic Regression on the Predictors of Mortality in COVID-19

**Parameters Percent Multiple Imputation by chained equations**

**Missing OR (95% CI) P value**

Reference 0.01 (0.004 - 0.03)

Age ≥ 60 years 0 2.01 (1.30 - 3.11) <0.01

Male 0 0.74 (0.47 - 1.15) 0.18

Hypertension 0 0.70 (0.46 - 1.08) 0.10

Diabetes mellitus 0 1.06 (0.68 - 1.65) 0.80

Heart Disease 0 1.10 (0.64 - 1.90) 0.73

COPD 0 2.34 (0.87 - 6.28) 0.09

Active tuberculosis 0 2.22 (0.91 - 5.39) 0.08

Chronic Kidney Disease 0 1.10 (0.59 - 2.06) 0.77

Neurologic Disease 0 1.40 (0.71 - 2.73) 0.33

Smoker 4.9 1.32 (0.81 - 2.13) 0.26

Shortness of breath 0 1.48 (0.97 - 2.25) 0.07

qSOFA score ≥ 2 0 8.23 (4.71 - 14.38) <0.01

White Blood Cell Count 3.5

< 4 x 10^9^/L 0.34 (0.09 - 1.22) 0.10

4 to 10 x 10^9^/L Ref

> 10 x 10^9^/L 1.59 (1.03 - 2.45) 0.03

Absolute lymphocyte count < 1000 3.6 1.86 (1.19 - 2.91) <0.01

Percent Neutrophil ≥ 70 3.6 2.39 (1.27 - 4.53) <0.01

Platelet count 3.8

< 100 x 10^9^/L 0.86 (0.28 - 2.66) 0.80

100 to 150 x 10^9^/L Ref

> 150 x 10^9^/L 0.43 (0.21 - 0.86) 0.02

PaO_2_/FiO_2_ Ratio 7.6

≤100 2.78 (1.41 - 5.47) <0.01

101-200 1.90 (1.11 - 3.25) 0.02

201-300 0.96 (0.54 - 1.70) 0.89

>300 Ref

eGFR < 90 5.3 1.78 (1.10 - 2.88) 0.02

Lactate dehydrogenase ≥ 600 U/L 8.2 2.43 (1.47 - 4.02) <0.01

Serum ferritin ≥ 600 5.3 1.28 (0.77 - 2.13) 0.33

C-reactive protein ≥ 12 mg/L 13.0 3.06 (1.53 - 6.11) <0.01

COPD – Chronic obstructive pulmonary disease; eGFR – estimated glomerular filtration rate; qSOFA – quick sequential organ failure assessment

*The CKD-EPI (Chronic Kidney Disease Epidemiology Collaboration) equation was used to estimate GFR


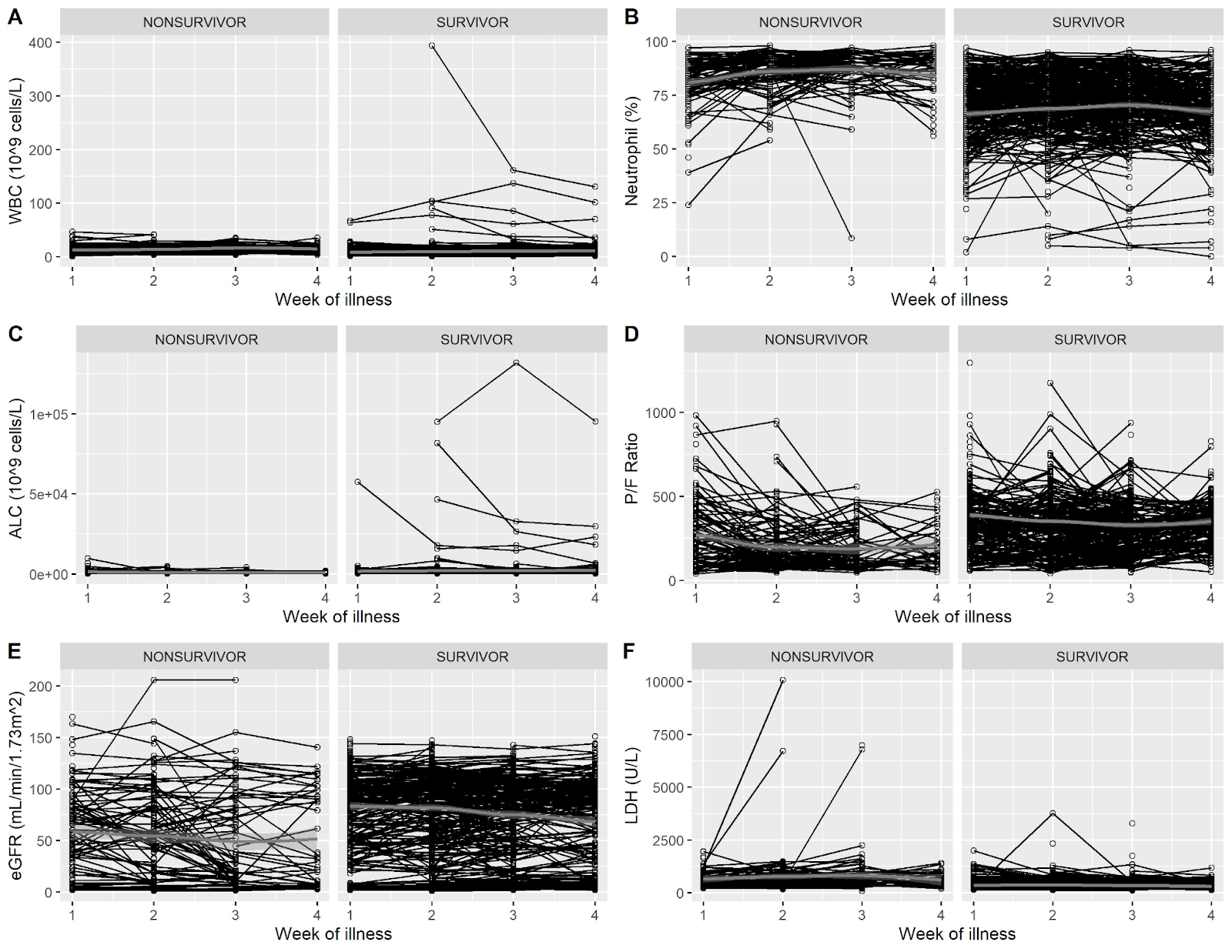


**Supplementary Figure 1.** Spaghetti plot of various laboratory parameters comparing COVID-19 survivors and nonsurvivors throughout the course of illness. Each black line represents a patient and weekly laboratory test results. Gray lines indicate mean values. A. White blood cell count; B. Percentage Neutrophil; C. Absolute lymphocyte count (ALC); D. PaO_2_/FiO_2_ ratio; E. estimated glomerular filtration rate (eGFR) calculated using CKD-EPI (Chronic Kidney Disease Epidemiology Collaboration) equation; F. Lactate dehydrogenase (LDH).
